# “ACHILLES” Heel No More? Afatinib at 40 Mg Once Daily is Superior to Platinum-Based Chemotherapy in *EGFR* Uncommon (G719X, S768I, and L861Q) Mutations (ACHILLES/TORG1834)

**DOI:** 10.2147/LCTT.S461758

**Published:** 2024-05-18

**Authors:** Faustine X Luo, Sai-Hong Ignatius Ou

**Affiliations:** 1 Chao Family Comprehensive Cancer Center, Orange, CA, 92868, USA; 2 University of California Irvine School of Medicine, Orange, CA, 92868, USA

**Keywords:** afatinib, uncommon EGFR mutations, G719X, S768I, L861Q, ACHILLES, relative dose intensity

## Abstract

Afatinib, a second-generation covalent EGFR TKI, has been approved for the treatment of the three “uncommon” *EGFR* mutations (G719X, S768I, and L861Q) based on one pooled retrospective analysis of three prospective trials (LUX-Lung 2, 3 and 6). The confirmed overall response rate, as assessed by independent radiology review, was 66% (95% confidence interval: 47–81). Among the 21 responders, the proportion of patients with response duration of ≥12 months was 52% and the proportion with response durations of ≥18 months was 33%. Of note, all patients received afatinib at 40 or 50 mg once daily which is higher than the approved dose of 40 mg once daily and the usual 30 mg once daily starting dose by most thoracic oncologists. Given the approval of afatinib for “uncommon” *EGFR* mutations was based on the limited number of patients analyzed, the retrospective nature of the analysis, lack of randomized phase 2 or 3 trial, there remains uncertainty as to whether afatinib, chemotherapy or other next-generation EGFR TKIs is the optimal treatment. This uncertainty also hinders the development of future treatment of these “uncommon” mutations because of the uncertainty that afatinib is the optimal treatment and hence should be the standard of care control arm in future randomized trials. Finally, the ACHILLES/TORG1834 provided us with the first randomized trial result that afatinib achieved superior progression-free survival over platinum-based chemotherapy (10.6 months vs 5.7 months, HR = 0.42; 95% CI: 0.256–0.694; P = 0.0007). However, ACHILLES should mostly be considered as phase 2 trial given the limited number (N = 109) of patients enrolled. Furthermore, the PFS benefit seemed to be with the 40 mg daily dose (HR = 0.128; 95% CI: 0.050–0.327) and not with the 30 mg daily dose (HR = 0.704; 95% CI: 0.352–1.406). Further investigation of the 30 once daily dosing for the treatment of uncommon *EGFR* mutations is needed.

## Introduction

The three “uncommon” epidermal growth factor receptor (*EGFR*) mutations (G719X, S768I, and L861Q) were discovered in the same year as the canonical activating *EGFR* mutations in 2004.^1^,^2^ Given the incidence of these three “uncommon” mutations are generally between 5% and 10% of the *EGFR* mutations,^3^ much more time and effort were devoted to develop the first-generation and then second-generation EGFR tyrosine kinase inhibitors (TKI) for the canonical *EGFR* mutations (del19 and L858R). Treatment for these three major “uncommon” *EGFR* mutations was put on the “backburner” for awhile.

Afatinib is a second generation covalent “irreversible” EGFR TKI that has inhibitory activity against both wildtype and canonical *EGFR* mutations (exon 19 deletion and L858R) activities was initially developed to overcome the acquired T790M mutation on progression from first-generation (1G) EGFR TKI. During the time of its initial clinical development, EGFR TKIs were compared to chemotherapy as first-line (1L) treatment of advanced metastatic *EGFR* mutations. The developmental program of afatinib (LUX-Lung) involved 8 trials with a global phase 2 trial of treatment-naïve NSCLC patients harboring all activating *EGFR* mutations (LUX-Lung 2)^4^ and two global phase 3 randomized trials against platinum-based chemotherapy (LUX-Lung 3 and LUX-Lung 6).^5^,^6^

The sponsor of afatinib, Boehringer Ingelheim, performed a retrospective analysis of the three above mentioned prospective trials that included 38 patients with any of the three “uncommon” *EGFR* mutations.^3^ Overall, the blinded independent central review (BICR) assessed ORR was 71.1%, (95% CI 54.1–84.6), median PFS 10.7 months (95% CI 5.6–14.7), and median overall survival was 19.4 months (95% CI 16.4–26.9).^3^ For each individual mutation, the ORR was 100% (8/8), median PFS of 14.7 months (95% CI: 2.6 - not reached) and median OS not reached for S768I. The ORR, median PFS and Median OS for G719X was 77.8% (14/18), 13.8 months 995% CI: 6.8 – not reached), and 26.9 months (95% CI: 16.4 months – not reached) respectively. The ORR, median PFS, and median OS was 56.3% (9/16), 8.2 months (95% CI: 4.6–16.6), and 17.1 months (95% CI: 15.3–21.6) respectively. On January 28, 2018, based on this analysis, afatinib received US FDA approval for the treatment of the three “non-resistant” “uncommon” *EGFR* mutations.^7^

Subsequently, Boehringer Ingelheim sponsored a large-scale pooled analysis from 15 studies (prospective trials, cohort studies, case series, case reports, patient assess programs) to further assess the clinical activity of afatinib in “uncommon” *EGFR* mutations. Given the heterogeneity of these studies, the common efficacy measure was time to treatment failure (TTF) defined as from to start to discontinuation of treatment. The median TTF for G719X patients (N = 69) was 14.7 months, 10.0 months for L861Q patients (N = 42), and 15.6 months for S768I patients. The aggregate data supported the efficacy of afatinib in the three uncommon mutations.^8^ However, TTF is a less vigorous endpoint than PFS and it is not defined in the paper how the TTF is calculated especially if there is an extended hold of afatinib due to side effects, whether the periods of dose interruptions are included in the TTF calculation. Additionally, treatment beyond progression will count towards TTF. Furthermore, despite the many more patients with uncommon mutations analyzed, the heterogeneity of the data source made this analysis less vigorous than a randomized trial.

## Design of ACHILLES/TORG1834

The ACHILLES/TORG1834 study is essentially a randomized phase II study (only 109 patients enrolled) conducted exclusively in Japan that compared afatinib versus platinum-doublet chemotherapy in patients with treatment-naïve advanced non-squamous NSCLC harboring any of the three “uncommon” *EGFR* mutations. The results were presented at the annual meeting of the European Society of Medical Oncology (ESMO) in 2023.^9^ Despite essentially the phase 2 design, there were 4 stratification factors: single versus compound uncommon *EGFR* mutations, stage III/IV versus recurrence, CNS metastasis (yes/no), and afatinib dose (30 mg versus 40 mg). Patients were randomized in a 2:1 ratio to afatinib 40 mg once daily (elderly/frail patients could start afatinib at 30 mg once daily) to either cisplatin 75 mg/m2 or carboplatin (AUC 5 or 6) and pemetrexed (500 mg/m2), followed by pemetrexed maintenance therapy every 3 weeks. The sample size of 106 was based on 75% power to detect a hazard ratio of 0.6 in PFS with α= 0.05.^9^

## Conduct of ACHILLES/TORG1834

One hundred and nine patients were enrolled over 4 years from Feb 2019 – Feb 2023 from 56 Japanese institutions. The median age of the patients on the afatinib arm was 71.0 years (range 49–83) old compared to 66.5 months (range 42–77) for patients who received chemotherapy. Patients were well balanced between the two arms. Thirty-seven patients received 30 mg of afatinib and 36 received 40 mg once daily dose. Of note, ~30% of the patients had brain metastasis and ~30% of the patients had compound “uncommon” mutations.

With a median follow-up time of 12.5 months (range 0–43.5) afatinib significantly improved progression-free survival (PFS) over platinum doublet chemotherapy. The median PFS was 10.6 months in the afatinib arm and 5.7 months in the chemotherapy arm (HR = 0.422; 95% CI: 0.256–0.694; p=0.0007). The event rate was 75.0% for afatinib and 79.4% for chemotherapy indicating maturity of the PFS values. The overall response rate was 61.4% for the afatinib arm and 47.1% for chemotherapy (P=0.2069). Subgroup analysis indicated afatinib was efficacious over chemotherapy in patients with or without brain metastasis and in patients with single or compound mutations. Importantly, the HR for 40 mg starting dose of afatinib was 0.128 (95% CI: 0.050–0.327) while HR for 30 mg starting dose of afatinib was 0.704 (95% CI: 0.352–1.406). Thus, the numerical difference in HRs between the 30 mg and 40 mg once daily was large. However, the actual PFS of each subgroup was not presented. Given the limited number of patients in the 30 mg and 40 mg starting dose and even fewer patients in the respective chemotherapy comparison arms due to 2:1 randomization and the wide 95% confidence interval. It remained to be determined if afatinib 30 mg starting dose is indeed inferior to 40 mg starting dose. We have to remember HR is a relative ratio and if the chemotherapy arm performed poorly among the elderly/frail patients who received 30 mg starting dose, it will important to understand the eventual PFS achieved by chemotherapy in the group of patients who were randomzied to the 30 mg start dose. Nonetheless, grade 3 or higher diarrhea occurred in 21.9% of the afatinib treated patients which is a significant morbidity for elderly patients. Other grade 3 or higher adverse events in afatinib arm included mucositis (8.2%) appetite loss (6.8%), and paronychia (6.8%). The duration of the adverse events is unknown given afatinib is taken daily and whether dose reduction resulted in lessening of the incidence and duration of these adverse events. The most common adverse events with chemotherapy were myelosuppression.

## Comments on ACHILLES/TORG1834

Given the limited number of patients enrolled into ACHILLES, it could only be at best described as a phase 2 trial despite the statistics being designed as a phase 3 trial. The 4 stratification factors included are all potential important prognostic factors for PFS outcome but excessive as it is rare to have 4 stratification factors even in large phase 3 randomzied trials such as LUX-Lung 3 or 6.^5^,^6^ Unbeknownst to a lot of clinicians, uncommon *EGFR* mutations sometimes occurred in tandem and the stratification for this observation was foresightful. The presence or absence of CNS metastasis is always an important differentiating factor in treatment outcome in all NSCLC. Comparing the 30 mg and 40 mg doses of afatinib will provide prospective data on the persisting question about the optimal dose of afatinib. Previous analysis indicated dose reduction from 40 mg to 30 mg did not affect efficacy.^10^ It will be important to compare the PFS acheived by afatinib 30 mg versus 40 mg and even more importantly the PFS acehvied by these two group of patients treated with chemotherapy as HR is a relative comparison. It is perceivable chemotherapy performed poorly (i.e. short PFS) in frail/elderly patients who received 30 mg starting dose. Nevertheless, including 4 stratification factors in such a small randomized patient population represent over-stratifications and the number of patients per stratum may not be sufficient to either detect a difference especially the control arm only contained 36 or 37 patients or exaggerated the differences due to limited number of patients in each subgroup.

## Concluding Comments

- ACHILLES trial provided us with some in-depth characteristics of *EGFR* patients with uncommon mutations. The most common mutation is G719X followed by L861Q. Single S768I mutation is rare. Compound mutations made up ~30% of the uncommon mutations. It will be important to decipher the molecular mechanism(s) that led to the compound mutations versus single mutation.
- The median PFS achieved by afatinib was 10.6 months essentially identical to the median PFS of 10.7 months reported in the retrospective analysis reported by Yang et al.^3^
- Afatinib 30 mg starting dose on subgroup analysis did not achieve a significant improved HR over chemotherapy. It seems all the beneficial effect came from the 40 mg dose. However, we have to recognize the very limited number of patients in the control arm (37 patients [30 mg] versus 19 patients [control]; 36 patients [40 mg] versus 17 patients [control]) and HR is a relative measure which depended on the performance of the chemtherapy comparison arm. We await if there is any bias in the background factors or efficacy data between the two groups especially among the patients who received chemotherapy (control).
- Given the limited number of patients in the 30 mg dose arm, the very wide 95% confidence interval. We will need to know the actual PFS numbers achieved by the afatinib 30 mg starting dose cohort compared to the 40 mg starting dose cohort from the eventual publication with comparison to the PFS acheived by chemotherapy within these two groups. Additionally, the time to dose reduction, the amount of dose reduction and the mean and median relative-dose intensity (RDI) was not reported in the presentation and hopefully will be presented in the eventual publication.
- The mean RDI of the 40 mg afatinib dose is important to report given the 40 mg patients may have dose reduced quickly and stayed at the 30mg dose. Importantly, mean RDI takes into account the “time” factor:-duration of dose interruptions and/or reductions.
- Of the 4 stratification factors employed in ACHILLES, from the results for advanced disease, stage (III/IV versus recurrence) and single or compound mutations could be deleted in future clinical trials. Given afatinib is approved for 40 mg, and the results of ACHILLES, 40 mg once daily should be the starting dose for afatinib in the control arm in future clinical trials. Thus, CNS metastasis and race will be the important prognostic factors to be stratified for in future global study of novel treatment strategy against afatinib.

## Remaining Questions to Be Followed by ACHILLES

- Is 30 mg still the appropriate starting dose for uncommon *EGFR* mutations commonly used by most community oncologists in the US? ACHILLES was a defacto a randomized phase 2 trial with 4 stratification factors which is a lot even for randomized phase 3 trials. Thus, subgroup analysis should be at best hypothesis-generating.

- What is the tolerability of the 40 mg starting dose given the median age of the patients are 71 years and grade 3 or higher diarrhea was 21.9%. The definition of grade 3 diarrhea is passing 7 or more stools a day than baseline. Grade 3 diarrhea may need treatment in a hospital or clinic. People with grade 3 diarrhea cannot control their bowel movements and have trouble meeting daily needs without help. It will be important to report the mean (especially) and median relative-dose intensity (RDI) which takes into account the duration and the magnitude of the dose reduction.
